# Analysis of Blood Culture Collection and Laboratory Processing Practices in Israel

**DOI:** 10.1001/jamanetworkopen.2022.38309

**Published:** 2022-10-25

**Authors:** Elizabeth Temkin, Dikla Biran, Tali Braun, David Schwartz, Yehuda Carmeli

**Affiliations:** 1 National Institute for Antibiotic Resistance and Infection Control, Israel Ministry of Health, Tel Aviv, Israel; 2 National Institute for Antibiotic Resistance and Infection Control, Israel Ministry of Health and Sackler Faculty of Medicine, Tel Aviv University, Tel Aviv, Israel

## Abstract

**Importance:**

Blood culturing is a critical diagnostic procedure affecting patient outcomes and antibiotic stewardship. Although there are standards for blood culturing, the process is not often measured.

**Objectives:**

To evaluate processes related to the diagnosis of bloodstream infection and compare them with best practices.

**Design, Setting, and Participants:**

A quality improvement study using laboratory data from January 1 to June 30, 2019, was conducted in 28 (96.6%) Israeli acute care hospitals. All blood cultures (BCs) performed on samples from adults and children in a period of 147 hospital-months were analyzed. Data analysis was performed from April 12, 2021, to September 9, 2022.

**Main Outcomes and Measures:**

True pathogen detection rate, contamination rate, proportion of adults with blood cultures performed, proportion of adult culturing episodes with only 1 set or bottle used, and median time of steps from sample collection to pathogen identification.

**Results:**

The data set consisted of 348 987 BC bottles. Bloodstream infection was detected in a median of 6.7% (IQR, 5.8%-8.2%) of adult culturing episodes and 1.1% (IQR, 0.7%-1.9%) of pediatric episodes. Eleven of 27 hospitals (40.7%) with adult patients met the standard of a contamination rate of less than 3% and only 2 hospitals (7.4%) met the more stringent standard of less than or equal to 1% contamination rate. The percentage of adults with blood cultures ranged from 2.7% to 29.0% (mean [SD], 15.7% [6.0%]). There was an association between sampling rate and pathogen detection until BCs were performed in 17% of adult admissions. The percentage of solitary BCs ranged from 47.8% to 94.4%. An estimated 1745 of 7436 (23.5%) adult bloodstream infections went undetected because solitary BCs were performed, anaerobic bottles were not used, or BCs were not performed. Median processing time was 51.2 (IQR, 33.9-78.0) hours, 3 times the optimal time: 4.4 (IQR, 1.7-12.5) hours for the preanalytical stage, 15.9 (IQR, 10.2-23.6) hours from incubation to growth detection, 4.5 (IQR, 1.5-10.7) hours from detection to Gram stain, and 30.9 (IQR, 22.0-41.9) hours from detection to isolate identification. An 8.6-hour delay was related to off-hours operating of laboratories.

**Conclusions and Relevance:**

The findings of this study suggest that the multistep process of blood culturing is not managed comprehensively in Israel, leading to poor clinical practices and delayed results.

## Key Points

**Question:**

Are blood cultures collected and processed in Israel in accordance with best practice standards?

**Findings:**

In this quality improvement study that included 28 hospitals (348 987 blood culture bottles), an estimated 24% of adult bloodstream infections went undetected because of undersampling of patients, use of solitary blood cultures, or lack of anaerobic bottles. Median time from blood draw to pathogen identification was 34 hours longer than best practice.

**Meaning:**

The findings of this study suggest that interventions to improve blood culturing processes are needed.

## Teaser

This quality improvement study examines the process of obtaining blood cultures for diagnosis of bloodstream infection, from sampling time to isolate identification in Israeli acute care hospitals.

## Introduction

Blood culturing, the most important diagnostic procedure in treating bacterial infections, comprises both clinical and laboratory processes and is one of the most essential tasks of the clinical microbiology laboratory.^1^ The primary goals are maximum detection of true pathogens with minimal contamination and speedy delivery of results. Delays in blood culture (BC) results may delay the initiation of appropriate antibiotic treatment, increasing the risk of death.^2,3^ Best practice standards have been proposed for various aspects of blood culturing^4,5,6,7^; because it is a multistep process involving clinical, ancillary, and laboratory staff across many departments, there are multiple opportunities for failure to meet these standards. Neither clinicians nor microbiologists are solely responsible for overseeing the blood culturing process. As a result, deficiencies in the process may go unnoticed or, if they are noticed, it is unclear whose job it is to correct them. The aim of this study was to evaluate processes related to the diagnosis of bloodstream infection (BSI) in Israeli hospitals and compare them with best practices to detect possible system weaknesses.

## Methods

### Data Collection

In this quality improvement study, data were collected as part of the Israeli Ministry of Health's infection control program. Since 2018, blood culturing practices and processes have been analyzed, with feedback to hospitals to promote improvement. In 2019, hospitals were asked to submit the following data on all BCs performed from January 1 to June 30, 2019: patient identification number; birth date; admission date; department; BC bottle type; and the dates and times of the blood draw, laboratory intake, start of incubation, growth detection, reporting of Gram stain results, reporting of pathogen, and reporting of antibiotic susceptibility testing (AST). The study was approved by the Tel Aviv Sourasky Medical Center Institutional Review Board. The informed consent requirement was waived for this analysis of deidentified data. This study followed the applicable portions of the Standards for Quality Improvement Reporting Excellence (SQUIRE) reporting guideline.

### Definitions and Inclusion Criteria

We defined a blood culturing episode as a 24-hour period.^8^ If blood draws continued for more than 24 hours, we considered them part of the same episode until there were no blood draws for 24 hours. For example, if blood samples for cultures were taken repeatedly over 72 hours with no 24-hour period without a blood draw, the 72 hours were considered as 1 episode. We defined a rapid test as organism identification within 4 hours of growth detection.^4^ We defined true pathogens and common commensals according to the National Healthcare Safety Network Organisms List.^9^ We considered common commensals to cause BSI if they were isolated at least twice on separate occasions.^10^ We defined common commensals isolated only once as contaminants; we applied this definition across all sites retrospectively. We classified patients as children if the BC was ordered in a pediatric department or the patient’s age was younger than 16 years.

We used the following criteria to overcome documentation errors: the start of incubation had to be 5 minutes to 72 hours after the blood draw. Reporting of Gram stain results had to occur after the positive signal and before reporting of isolate identification. Isolate identification had to occur after Gram stain reporting and within 120 hours of the positive signal. To determine conformity to best practices at the hospital level, we included only hospitals with informative data, defined as having plausible documentation for at least half of all bottles.

### Statistical Analysis

Data analysis was performed from April 12, 2021, to September 9, 2022. Hospitals were categorized into group 1 (>700 beds or tertiary care), group 2 (400-700 beds), or group 3 (<400 beds). We used a *t* test or analysis of variance to compare means, Wilcoxon rank sum or Kruskal-Wallis to compare medians, and χ^2^ to compare proportions.

To determine the minimum proportion of adult admissions in which BC should be performed (sampling rate) to prevent underdetection of BSI, we used data from 2018-2020. We used a segmented generalized estimating equation with the proportion of admissions with BSI detected as the outcome and sampling rate, modeled as a spline, as the predictor. We chose as the spline knot (ie, the optimal minimum sampling rate) the value that produced the steepest slope below the knot and a nonsignificant, nonnegative slope above the knot.

To determine whether the number of BC sets obtained per episode reflected confounding by indication (ie, only 1 set was performed because there was a low suspicion of BSI), we compared the proportion of first sets positive for a true pathogen when additional sets were not obtained vs when they were obtained. We expressed these 2 values as a positivity ratio. To estimate the number of BSIs that went undetected because no anaerobic bottle was used, we multiplied the percentage of true pathogens detected only in anaerobic bottles by the number of episodes with no anaerobic bottles. To estimate BSI that went undetected because only 1 aerobic bottle was taken, we multiplied the percentage of episodes with a true pathogen detected only in subsequent aerobic bottles by the number of negative episodes with single aerobic bottles. Then, to account for the possibility that single bottles were collected in episodes with lower risk of a pathogen in additional bottles, we multiplied this result by the positivity ratio.

## Results

### Data Selection

Twenty-eight of 29 eligible hospitals (96.6%) contributed 147 hospital-months of data: 8 in group 1 (including 1 children's hospital), 9 in group 2, and 11 in group 3. The data set consisted of 348 987 BC bottles: 289 974 (83.1%) from adults and 59 013 (16.9%) from children. The hospitals' microbiology laboratories are described in the eMethods in the Supplement.

### Culture Results

By hospital, median positivity of BC bottles taken from adults was 8.9% (IQR, 7.2%-11.3%). Median positivity with a true pathogen was 5.4% (IQR, 4.9%-6.8%), and median positivity with only a contaminant was 3.1% (IQR, 2.1%-4.4%). Eleven (40.7%) of the 27 hospitals with adult patients met the Public Health England (PHE) standard of a contamination rate less than 3%^5^; 2 hospitals (7.4%) met the suggested revised standard of less than or equal to 1%.^6^ Among children, median positivity was 3.6% (IQR, 2.4%-4.4%) overall, 1.2% (0.7%-2.1%) for true pathogens, and 2.2% (1.5%-2.5%) for contaminants only.

**Figure 1.**
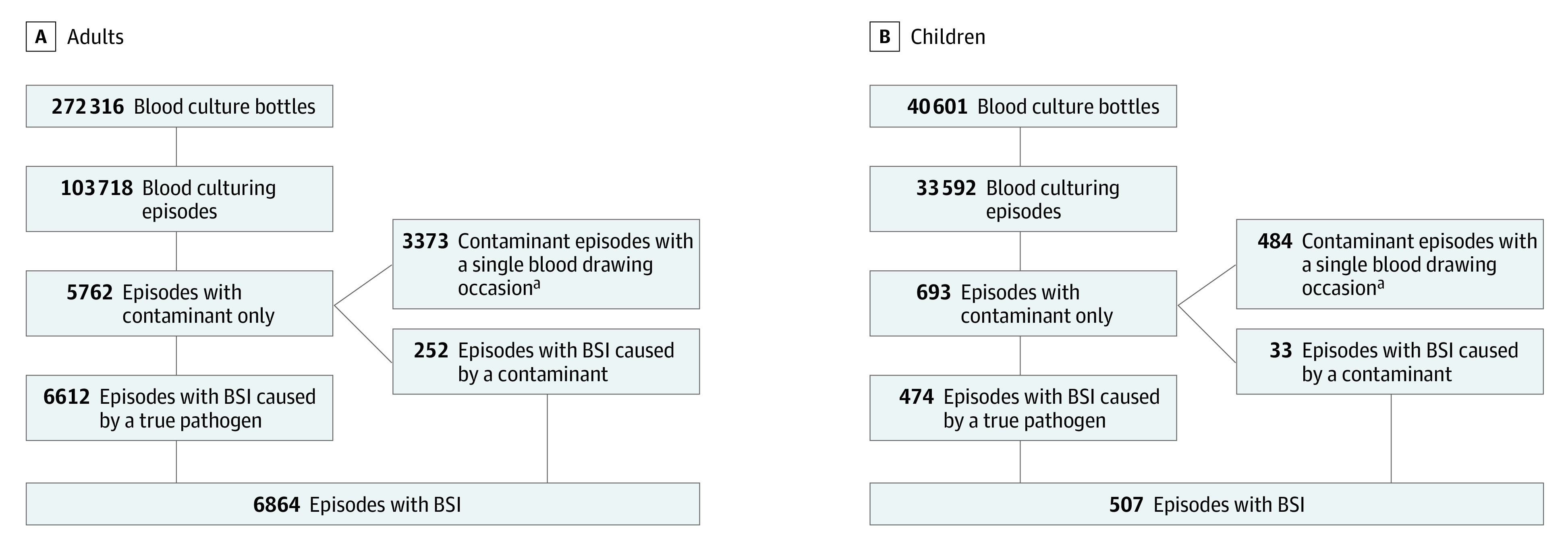
Positivity of Blood Culturing Episodes Among Adults and Children BSI indicates bloodstream infection. ^a^Single blood sampling occasion: 1 blood draw per episode or 2 blood draws less than 2 minutes apart and none the following day (ie, no opportunity to detect BSIs caused by a common commensal).

There was sufficient documentation for grouping 272 316 (93.9%) of adult bottles and 40 601 (68.8%) of bottles from children into blood culturing episodes. Detection of BSIs and contaminants is shown in Figure 1. By hospital, BSIs were detected in a median of 6.7% (IQR, 5.8%-8.2%) of adult episodes and only a contaminant was detected in a median of 4.8% (IQR, 3.8%-6.7%). Bloodstream infections were detected in a median of 1.1% (IQR, 0.7%-1.9%) of pediatric episodes and only a contaminant was detected in a median of 2.1% (IQR, 1.4%-2.4%) of pediatric episodes. Among adults, the contamination rate was highest in general intensive care units (8.3%; IQR, 5.7%-11.0%).

### Frequency of Collecting Blood Culture Samples

Among adults, the number of BC bottles per 100 patient-days ranged widely between hospitals, from 2.5 to 25.8. The mean (SD) rate was 15.5 (6.7) and did not differ significantly between hospital groups (*P* = .48). The percentage of adult admissions in which at least 1 BC was performed ranged from 2.7% to 29.0%, with a mean (SD) of 15.7% (6.0%) and no significant difference between hospital groups (*P* = .43).

We found an association between sampling rate and pathogen detection until samples for BCs were obtained in 17% of adult admissions (eFigure in the Supplement). For each additional 1% of admissions in which samples for BC were obtained, the percentage in which a BSI caused by a true pathogen was detected increased by 0.09% (95% CI, 0.08%-0.11%; *P* < .001). Above 17% of admissions, the finding was no longer significant (β = 0.02%; 95% CI, −0.01% to 0.05%; *P* = .14). Bloodstream infection caused by a true pathogen was detected in a mean (SD) of 1.7% (0.4%) admissions when BCs were performed in 17% or more admissions. We estimated that if hospitals with lower sampling rates had performed BCs in 17% of admissions, admissions in which a BSI was detected would have increased nationwide by 15.7% (95% CI, 3.4%-32.7%) from 6910 to 7998 (95% CI, 7148-9167).

### Sets per Episode and Use of Anaerobic Bottles

Among 103 718 adult blood culturing episodes, in 72 670 episodes (70.1%), no additional sets or no additional aerobic bottles were obtained within 24 hours (solitary BC). Solitary BCs were most common in surgical wards (79.2%) and least common in internal medicine wards (65.4%). By hospital, the percentage of solitary BCs ranged from 47.8% to 94.4%. No hospital met the standard proposed by Lamy et al^7^ of less than 10% solitary BCs. Counting individual bottles (not sets) taken from adults, 2 bottles were used in 67 142 (64.7%) episodes, 4 bottles in 19 106 (18.4%) episodes, and 1 bottle in 9491 (9.2%) of episodes. Of these single bottles, 9048 (95.3%) were aerobic.

Among 92 817 episodes (89.5%) with a recorded admission date, median time from admission to blood culture was 1 day (IQR, 0-4 days). Solitary BCs were more common for the diagnosis of hospital-acquired BSI: solitary BCs were performed in 41 634 of 63 346 episodes (65.7%) within the first 3 days and in 22 149 of 29 471 episodes (75.2%) after day 3 (*P* < .001). Detection of a true pathogen was higher in multiset episodes (3311 of 31 048 [10.7%]) than in solitary BC episodes (3301 of 72 670 [4.5%]) (*P* < .001). This difference in detection rate remained significant even when considering only the first set obtained in multiset episodes: true pathogens were detected in 8.4% of first sets in episodes with additional sets (*P* < .001 vs 4.5% detection rate in solitary BCs).

The median ratio of anaerobic to aerobic bottles was 1.0 (IQR, 0.92-1.0); in 4 hospitals the ratio was less than 0.60. The distribution of the 10 leading pathogens isolated in aerobic and anaerobic bottles is reported in the eTable in the Supplement; *Escherichia coli* was most common, accounting for 28.3% of pathogens isolated in aerobic bottles and 30.3% of pathogens isolated in anaerobic bottles. When aerobic and anaerobic bottles were obtained at the same time, 20.7% of the pathogens were detected in anaerobic bottles only (Table 1).

**Table 1.** Pathogens Detected Only in Anaerobic Bottles When Aerobic-Anaerobic Sets Were Collected

Variable	Positive sets, No.	Sets with only anaerobic bottle positive, No. (%)
All pathogens	8544	1765 (20.7)
*Bacteroides* spp	210	202 (96.2)
*Clostridioides* spp	64	53 (82.8)
*Morganella morganii*	83	25 (30.1)
*Proteus* spp	372	91 (24.5)
*Escherichia coli*	2197	450 (20.5)
*Klebsiella pneumoniae*	833	171 (20.5)
*Enterococcus faecalis*	577	106 (18.4)
*Staphylococcus aureus*	1504	220 (14.6)
*Candida* spp	210	14 (6.7)
*Pseudomonas aeruginosa*	482	29 (6.0)
*Acinetobacter* spp	206	4 (1.9)

Based on data from a subsample of 25 hospitals that serve adults and reported by bottle type, we estimated that if at least 2 aerobic-anaerobic sets were obtained per episode, and if samples for BC were drawn in at least 17% of adult admissions, then detection of BSI by a true pathogen would increase by 31% (from 5691 to 7436). We estimated that 23.5% (1745 of 7436) of adult BSIs were missed: 542 (7.3%) because of using a single aerobic bottle, 193 (2.6%) because of not using an anaerobic bottle, and 1009 (13.6%) because of undersampling.

### Documentation

Sample collection dates and times were documented for 269 730 (77.3%) of BC bottles. Only 209 155 bottles (59.9%) had documented plausible dates and times of both collection and start of incubation. By hospital, documentation ranged from 0.0% to 98.5% (median, 75.1%). Among 25 081 positive bottles and an additional 2068 positive sets (from 2 hospitals that did not indicate whether 1 or both bottles were positive), the dates and times of the positive signal, Gram stain reporting, and isolate identification were documented and plausible for 16 642 (61.3%). By hospital, plausible documentation for positive bottles ranged from 0.0% to 99.1% (median, 79.2%).

### Processing Times

**Figure 2.**
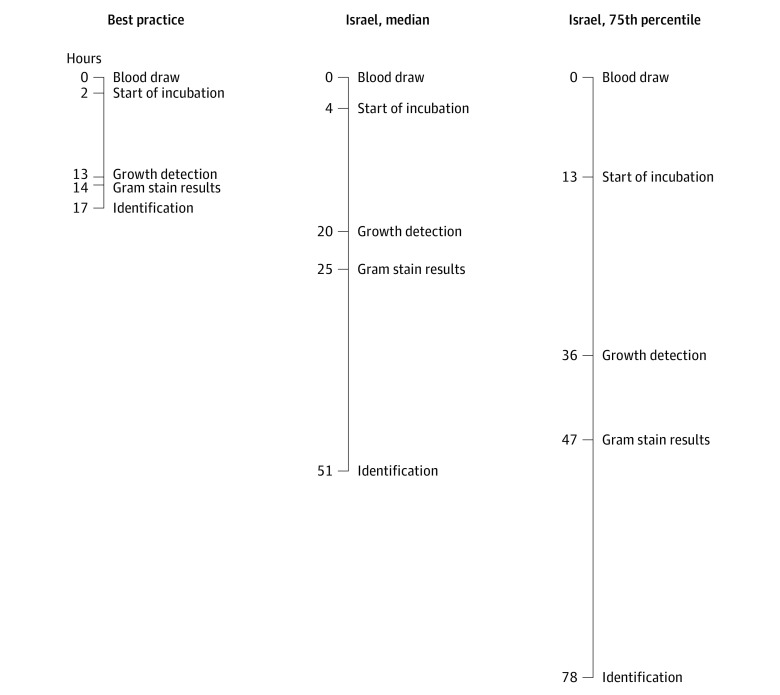
Timing of Laboratory Processes: Comparison of Study Median and 75th Percentile in Israel With Best Practices

Table 2 summarizes BC processing times and Figure 2 compares the times with best practices. Median processing time was 51.2 (IQR, 33.9-78.0) hours: 4.4 (IQR, 1.7-12.5) hours for the preanalytical stage, 15.9 (IQR, 10.2-23.6) hours from incubation to growth detection, 4.5 (IQR, 1.5-10.7) hours from detection to Gram stain, and 30.9 (IQR, 22.0-41.9) hours from detection to isolate identification. Of 209 155 bottles, 60 528 (28.9%) met the best practices standard of a preanalytical stage less than or equal to 2 hours^4^ and 99 140 (47.4%) met the PHE standard of less than or equal to 4 hours.^5^ Of 16 hospitals with informative data, 3 had a median preanalytical stage of less than or equal to 2 hours and 6 had a median of less than or equal to 4 hours.

**Table 2.** Duration of Stages in Blood Culture Processing

Variable	Median (IQR), h			
	Preanalytical stage	Time to detection^a^	Growth detection to reporting of Gram stain results	Growth detection to identification
All BC bottles^b^	4.4 (1.7-12.5)	15.9 (10.2-23.6)	4.5 (1.5-10.7)	30.9 (22.0-41.9)
Start of step^c^				
Weekday, day shift (8 am to 8 pm), nonholiday	3.0 (1.5-9.3)	NR	1.6 (0.8-3.5)	23.7 (19.3-40.4)
Other	7.3 (2.3-12.7)	NR	7.0 (3.0-11.4)	33.0 (26.3-44.2)
Hospital group^c^				
>700 beds or tertiary care	3.6 (1.5-9.7)	NR	3.2 (1.2-8.1)	28.2 (20.4-39.4)
400-700 beds	10.7 (3.2-16.7)	NR	8.0 (2.4-14.0)	33.4 (25.2-41.6)
<400 beds	2.5 (1.0-8.1)	NR	3.3 (1.4-8.8)	31.3 (20.6-49.6)

Abbreviation: NR, not reported.

^a^ Time to detection depends primarily on blood volume, which we did not measure.

^b^ Includes only bottles with plausible, documented times: for the preanalytical stage, n = 209 155; for other stages, n = 16 642 (positives only).

^c^ *P* < .001 for all comparisons between weekday days vs other times and between hospital groups.

Among 16 642 positive bottles, median time to detection was 15.9 hours (IQR, 10.2-23.6 hours), compared with the optimal practice of 10.3 hours, as reported in a study using the BacT/Alert 3D automated system.^11^ None of the 19 hospitals with informative data achieved a median time to detection of 10.3 hours.

Median time from growth detection to reporting of Gram stain results was 4.5 hours (IQR, 1.5-10.7 hours), compared with the best practice of 1 hour or less^12^ and the PHE goal of less than or equal to 2 hours to reporting of Gram stain results.^5^ Median time from growth detection to Gram stain was less than or equal to 2 hours in 3 of 19 hospitals.

Median time from growth detection to isolate identification was 30.9 hours (IQR, 22.0-41.9), compared with the optimal practice of 4 hours using rapid testing methods.^4^ The PHE set a goal of 24 hours^5^; 30.4% of bottles met this standard. Only 1 of 19 hospitals identified at least half of all isolates within 24 hours. Rapid testing was performed on 0.6% of positive bottles or 0.8% of positive episodes. Identification within 4 hours of Gram stain results occurred for 7.5% of positive bottles.

The duration of all steps dependent on laboratory staff (ie, all but time to detection) met the PHE standards when performed on the day shift on nonholiday weekdays. An 8.6-hour delay in median time from blood sample draw to identification was related to off-hours operating of laboratories. We could not summarize data on time to antibiotic susceptibility results, because results for different antibiotics were reported at different times and the single time listed was not necessarily for the most relevant agents.

## Discussion

In this report that included 96.6% of Israeli hospitals, we found major deficiencies in obtaining and processing of blood cultures. We estimated that almost a quarter of all BSIs went undetected because of undersampling of patients, obtaining solitary BCs, or lack of anaerobic cultures. Contamination rates were much higher than the recommended threshold. We found delays at all steps, with a median time from blood draw to identification that was 3 times longer than best practices, leading to a 34-hour delay in pathogen identification. Processing times were long despite the fact that more than 90% of laboratories are equipped with automated BC systems and matrix-assisted laser desorption/ionization-time of flight systems. Longer processing times at night and on weekends indicated inadequate laboratory staffing and practices in off-hours. Documentation of dates and times was poor, suggesting that BC processes are not monitored or managed, hindering quality improvement efforts. Culturing practices varied widely between hospitals, with a 10-fold difference in the number of BCs performed per 100 adult patient-days.

Previous studies of BC practices have taken 2 forms: surveys and, as in our study, analysis of laboratory data. A 2016-2017 survey of 209 laboratories in 25 European countries revealed weaknesses: at least 2 BC sets were submitted consistently in only 40% of laboratories, only 42% had the capacity to start incubation at any time, and only 13% had 24-hour service to allow immediate processing of bottles that flagged positive.^13^ A US study of laboratory data on more than 165 000 BCs from 13 hospitals in 2015-2016 found turnaround times that were longer than best practice goals: a median of 20.4 hours from collection to Gram stain and 43.9 hours from blood draw to isolate identification.^14^ In contrast, another analysis of 36 US hospital laboratories (n = 1586 positive BCs) in 2016 demonstrated that best practice goals are within reach: median preanalytical time was 47 minutes and median time from blood draw to identification was 16.4 hours.^15^

We studied 3 practices that could lead to underdetection of BSIs. The first was performing BCs in too few patients. We determined that BSI was likely underdetected in hospitals that performed BCs in less than 17% of adult patients. A study in 223 German intensive care units found a pattern similar to ours: BSI detection increased as the sampling rate increased until reaching a saturation point (80-90 BC sets per 1000 patient-days), after which there was no association between sampling rate and detection.^16^ These benchmarks are likely not generalizable to other settings, as they will vary with BSI incidence, which depends on factors such as case mix, antibiotic resistance levels, and infection control practices. We lacked *International Classification of Diseases* admission codes, which would have allowed us to control for case mix. Friedman et al^17^ suggested that BC positivity may be a more useful quality indicator than sampling rate; we did not use this indicator, because it is influenced by blood volume (data that we did not have) and the number of BC sets obtained.

The second reason for underdetection was solitary BCs. We estimated that 7.3% of BSIs were missed because only a single aerobic bottle was obtained. An analysis performed in 1983 found 10% underdetection of BSIs by the first BC set compared with 2 sets.^18^ More recently, in a study of patients from whom at least 3 sets were collected, 27% of BSIs were detected after the first set.^8^ In our study, the paucity of episodes with multiple BCs and the lack of data on blood volume limited our ability to discern the true yield of subsequent bottles.

The third cause of underdetection was failure to perform anaerobic BCs. Although the ratio of aerobic to anaerobic bottles was close to 1.0 in most hospitals, we estimated that 3% of BSIs went undetected because an anaerobic bottle was not used. One British hospital tried collecting aerobic bottles only, hypothesizing that greater blood volume in a single bottle would enhance detection more than adding an anaerobic bottle. The practice was abandoned after finding higher pathogen detection in the control hospital collecting aerobic-anaerobic pairs.^19^

Although we identified aspects of insufficient sampling, there also appears to be oversampling or indiscriminate sampling. One sign of this poor diagnostic stewardship is the low positivity rate^20^: true pathogens were detected in a median of 6.7% of BC bottles in adults and a median of 1.1% of BC bottles in children. A second sign was our finding of lower positivity among solitary BCs than in first sets in episodes with multiple BCs performed, suggesting confounding by indication, ie, only 1 set was obtained because there was a low suspicion of BSI. The practice of reflexive culturing of patients at low-risk may stem from the lack of expert guidelines delineating indications for drawing samples for BC.^21^ Another possible explanation is that the negligence reflected in performing a solitary BC is coupled with drawing a smaller volume of blood, which results in lower positivity.

Interventions can correct the weaknesses identified in this study. During sample collection, decision support reminders in the electronic medical record can reduce solitary BCs.^22^ Contamination can be reduced by initial specimen diversion,^23^ using dedicated phlebotomists,^24^ and avoiding blood draws from intravenous catheters.^25^ Blood culture positivity can be increased by increasing the volume of blood collected through education and feedback for personnel, marking the 10-mL point on bottles,^26^ and supplying clinical decision tools to avoid BCs in patients at low risk of BSI.^20^ The preanalytical stage can be shortened by staffing the microbiology laboratory 24 hours per day^27^ or by operating incubators in other laboratories with 24-hour service^28^ or at the point of care.^29^ Strategies to shorten the analytical stage include ensuring adequate blood volume,^15^ performing Gram stains in the core laboratory when the microbiology laboratory is closed,^12^ and adopting rapid testing technology. In a meta-analysis, molecular rapid diagnostic testing was reported to decrease mortality following BSI by 34%, translating into the need to use rapid testing on 20 patients with BSI to prevent 1 death.^30^

### Limitations

This study has limitations. First, although we excluded samples with illogical documentation, our results may partially reflect documentation errors rather than actual practice. Second, our calculations of processing times included only bottles with proper documentation; they may not be representative of all BCs. Third, we lacked data on blood volume, which is an important variable affecting BC positivity and time to detection. Fourth, we lacked data on whether blood samples for cultures were drawn before or after antibiotics were given. Drawing blood before antibiotics are given is an important element of proper blood culturing practice. Drawing blood after antibiotic therapy has started could contribute to the low culture positivity that we observed.

## Conclusions

Our study highlights the complexity and pitfalls of blood culturing. The findings suggest that the blood culturing process is inadequately monitored and managed, resulting in poor detection of BSI and delayed results. The blood culturing process should be continuously monitored and improved through data collection, analysis, and intervention.

## Notes

Supplement. **eMethods.** Description of Microbiology Laboratories in Participating Hospitals
eReference
**eTable.** Ten Most Common True Pathogens Isolated in Adult Blood Cultures, by Bottle Type **eFigure.** Association Between the Percentage of Adult Hospital Admissions With Blood Cultures Taken and the Percentage of Adult Admissions in Which BSI Caused by a True Pathogen Was Detected
