# Supplementary material for: Analysis of Blood Culture Collection and Laboratory Processing Practices in Israel
Source: JAMA Netw Open. 2022 Oct 25;5(10):e2238309. doi: 10.1001/jamanetworkopen.2022.38309 (PMC9597385; doi:10.1001/jamanetworkopen.2022.38309)
Supplement: Supplement. — eMethods. Description of Microbiology Laboratories in Participating Hospitals eReference eTable. Ten Most Common True Pathogens Isolated in Adult Blood Cultures, by Bottle Type eFigure. Association Between the Percentage of Adult Hospital Admissions With Blood Cultures Taken and the Percentage of Adult Admissions in Which BSI Caused by a True Pathogen Was Detected [file jamanetwopen-e2238309-s001.pdf]

Supplementary Online Content

Temkin E, Biran D, Braun T, Schwartz D, Carmeli Y. Analysis of blood culture collection and laboratory processing practices in Israel. *JAMA Netw Open*. 2022;5(10):e2238309. doi:10.1001/jamanetworkopen.2022.38309

eMethods. Description of Microbiology Laboratories in Participating Hospitals

eReference

eTable. Ten Most Common True Pathogens Isolated in Adult Blood Cultures, by Bottle Type

eFigure. Association Between the Percentage of Adult Hospital Admissions With Blood Cultures Taken and the Percentage of Adult Admissions in Which BSI Caused by a True Pathogen Was Detected

This supplementary material has been provided by the authors to give readers additional information about their work.

© 2022 Temkin E et al. *JAMA Network Open*.

eMethods

Description of microbiology laboratories in participating hospitals

All 28 participating hospitals have a microbiology lab on site. (The one dedicated children's hospital shares a lab with the adjacent hospital that serves adults and newborns.) All labs are run by a dedicated microbiologist. All hospitals have an on-call infectious disease service available 24 hours. No hospitals have a blood culture service such as the one described by Fitzpatrick et al., in which a microbiologist confers with medical and nursing staff every evening on new positive blood cultures.[1] All labs use automated blood culture systems: 18 use BD BACTEC (Becton, Dickinson and Company), 7 use BACT/ALERT VIRTUO (bioMérieux), and 2 use BACT/ALERT (bioMérieux). Only one lab operates 24 hours a day. The other labs are fully staffed from 8:00 a.m. to 4:00 p.m. and minimally staffed to perform urgent tests (including blood cultures) until 10:00 p.m. Labs in large and mid-sized hospitals are minimally staffed to perform urgent tests on Saturdays and holidays. All labs either are accredited by the College of American Pathologists (CAP) or undergo external quality assessment by UK NEQAS. All labs are certified by the Israeli Ministry of Health and are inspected periodically by a microbiologist from the National Institute for Antibiotic Resistance and Infection Control.

eReference

1. Fitzpatrick F, Turley M, Humphreys H, Smyth E. An after-hours clinical liaison blood culture service--is it worth it? *Clin Microbiol Infect*. 2004;10(10): 917-21. doi:10.1111/j.1469-0691.2004.00914.x

© 2022 Temkin E et al. *JAMA Network Open*.

eTable. Ten Most Common True Pathogens Isolated in Adult Blood Cultures, by Bottle Type.[1]

Aerobic bottles (n=4888)		Anaerobic bottles (n=4059)	
**Pathogen**	**n (%)**	**Pathogen**	**n (%)**
Escherichia coli	1384 (28.3)	*Escherichia coli*	1229 (30.3)
Staphylococcus aureus	609 (12.5)	*Staphylococcus aureus*	544 (13.4)
Klebsiella pneumoniae	512 (10.5)	*Klebsiella pneumoniae*	469 (11.6)
Pseudomonas aeruginosa	334 (6.8)	*Enterococcus faecalis*	282 (6.9)
Enterococcus faecalis	313 (6.4)	*Proteus mirabilis*	221 (5.4)
Proteus mirabilis	231 (4.7)	*Bacteroides fragilis*	118 (2.9)
Acinetobacter baumannii	122 (2.5)	*Streptococcus pneumoniae*	106 (2.6)
Streptococcus pneumoniae	122 (2.5)	*Pseudomonas aeruginosa*	101 (2.5)
Enterococcus faecium	84 (1.7)	*Enterobacter cloacae*[2]	91 (2.2)
Enterobacter cloacae[2]	77 (3.1)	*Enterococcus faecium*	69 (1.7)
Other	1096 (22.4)	Other	829 (20.4)

[1]Pathogens isolated repeatedly from the same patient during the same hospitalization were counted once. The analysis excludes 3 hospitals that did not report or did not consistently report bottle type.
[2]Includes *Enterobacter cloacae/asburiae* and *Enterobacter cloacae* complex

© 2022 Temkin E et al. *JAMA Network Open*.

eFigure. Association Between the Percentage of Adult Hospital Admissions With Blood Cultures Taken and the Percentage of Adult Admissions in Which BSI Caused by a True Pathogen Was Detected. Segmented generalized estimating equation with spline knot at 17% of admissions.

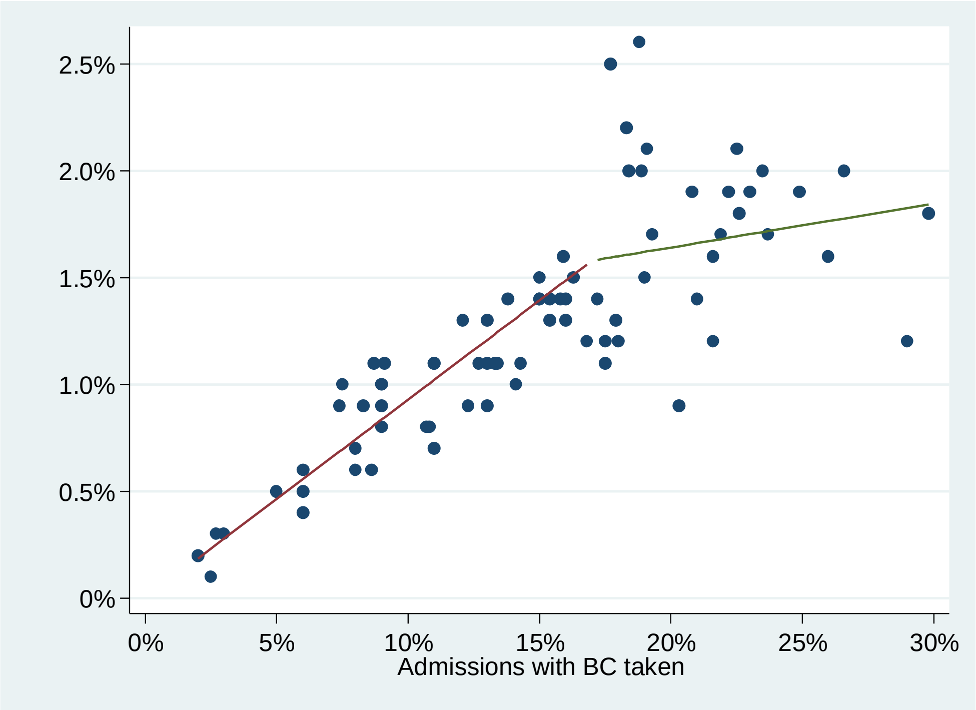

© 2022 Temkin E et al. *JAMA Network Open*.
